# Cullin-5 neddylation-mediated NOXA degradation is enhanced by PRDX1 oligomers in colorectal cancer

**DOI:** 10.1038/s41419-021-03557-3

**Published:** 2021-03-12

**Authors:** Shoufang Xu, Yilei Ma, Qingchao Tong, Jun Yang, Jia Liu, Yanzhong Wang, Guoli Li, Jin Zeng, Sining Fang, Fengying Li, Xinyou Xie, Jun Zhang

**Affiliations:** 1 Department of Clinical Laboratory, Sir Run Run Shaw Hospital, School of Medicine, Zhejiang University, Hangzhou, Zhejiang P.R. China; 2 Key Laboratory of Biotherapy of Zhejiang Province, Hangzhou, Zhejiang P.R. China; 3 Department of Cytopathology, Ningbo Diagnostic Pathology Center, Ningbo, Zhejiang P.R. China; 4 Ningbo Medical Center Lihuili Hospital, Ningbo, Zhejiang, P. R. China

**Keywords:** Colorectal cancer, Apoptosis, Neddylation, Ubiquitylation

## Abstract

NOXA, a BH3-only proapoptotic protein involved in regulating cell death decisions, is highly expressed but short-lived in colorectal cancer (CRC). Neddylated cullin-5 (CUL5)-mediated ubiquitination and degradation of NOXA is crucial to prevent its overaccumulation and maintain an appropriate action time. However, how this process is manipulated by CRC cells commonly exposed to oxidative stress remain unknown. The peroxiredoxin PRDX1, a conceivable antioxidant overexpressed in CRC tissues, has been shown to inhibit apoptosis and TRAF6 ubiquitin-ligase activity. In this study, we found that PRDX1 inhibits CRC cell apoptosis by downregulating NOXA. Mechanistically, PRDX1 promotes NOXA ubiquitination and degradation, which completely depend on CUL5 neddylation. Further studies have demonstrated that PRDX1 oligomers bind with both the Nedd8-conjugating enzyme UBE2F and CUL5 and that this tricomplex is critical for CUL5 neddylation, since silencing PRDX1 or inhibiting PRDX1 oligomerization greatly dampens CUL5 neddylation and NOXA degradation. An increase in reactive oxygen species (ROS) is not only a hallmark of cancer cells but also the leading driving force for PRDX1 oligomerization. As shown in our study, although ROS play a role in upregulating *NOXA* mRNA transcription, ROS scavenging in CRC cells by N-acetyl-L-cysteine (NAC) can significantly reduce CUL5 neddylation and extend the NOXA protein half-life. Therefore, in CRC, PRDX1 plays a key role in maintaining intracellular homeostasis under conditions of high metabolic activity by reinforcing UBE2F-CUL5-mediated degradation of NOXA, which is also evidenced in the resistance of CRC cells to etoposide treatment. Based on these findings, targeting PRDX1 could be an effective strategy to overcome the resistance of CRC to DNA damage-inducing chemotherapeutics.

## Introduction

Colorectal cancer (CRC) accounts for ~10% of all diagnosed cancers annually and is currently the fourth most deadly cancer worldwide^1,2^, the continuous emergence of drug resistance and heterogeneity remain major challenges for CRC treatment^3,4^. Defects in apoptosis are hallmarks of cancer cells^5^, and cancer cells exploit various apoptosis escape strategies to overcome normal growth constraints and acquire resistance to chemotherapeutics^6,7^. In the process of B cell lymphoma 2 (BCL-2) protein-mediated mitochondrial apoptosis^8,9^, BH3-only proteins act as stress sentinels that relay diverse upstream apoptotic signals to mitochondria, leading to mitochondrial outer membrane permeabilization and apoptosis^10,11^.

NOXA is a member of the BH3-only proteins and plays a vital role in the cellular response to anticancer agents^12^. NOXA performs its proapoptotic function mainly by selectively neutralizing the pro-survival BCL-2 family protein MCL1/A1, thereby facilitating the activation of BAX/BAK proteins^13^. To date, both p53-dependent and p53-independent regulation of *NOXA* transcription have been reported under various stress conditions, including DNA damage, hypoxia, mitogenic stimulation, cytokine signaling (IL-7/IL-15) and ER stress^14^. In addition, general upregulation of NOXA has been observed in various normal and malignant tissues^12,13^. To maintain their high proliferative potential and evade anticancer therapies, cancer cells have developed strategies to counteract the effects of increased *NOXA* transcription. It has been established in both lymphocytic leukemia and lung cancer that NOXA is a short-lived protein (with a half-life of less than 2 h) and undergoes K11-linked polyubiquitination mediated by the CUL5-RING-ligase (CRL5) complex^15,16^. Proteasome inhibition-induced apoptosis of lung cancer and hematopoietic cells is associated with accumulation of NOXA^17,18^. Therefore, ubiquitin-proteasome system (UPS)-mediated degradation of NOXA is critical for its rapid removal, but the mechanism by which this process is coordinated in cancer cells remains largely unknown.

PRDX1, a typical 2-Cys peroxiredoxin, was first reported to be an important endogenous antioxidant, protecting cells from oxidative damage by reducing reactive oxygen species (ROS) and peroxynitrite levels and scavenging thiyl radicals^19^. In addition to its antioxidant activity, PRDX1 functions as a chaperone in the form of a high molecular weight (HMW) complex and exhibits these molecular chaperone activities under oxidative stress condition^20,21^. Accumulating evidence shows that these oligomeric forms of PRDX1 can directly bind with a variety of proteins, thus affecting their bioactivities, participating in signal transduction essential for cell differentiation, proliferation and apoptosis^22,23^. Min et al.^24^ reported that PRDX1 inhibits TRAF6 ubiquitin-ligase activity. However, whether PRDX1 participates in ubiquitination pathway associated NOXA degradation or whether PRDX1 inhibits NOXA-associated apoptosis and the mechanisms by which it does so remain unknown.

In this study, we found that NOXA is a highly expressed but short-lived protein in CRC. PRDX1 shows a negative correlation with the NOXA protein half-life and protects CRC cells from apoptosis by enhancing NOXA ubiquitination and degradation. This effect arises because PRDX1 specifically potentiates CUL5 neddylation, which is the key to activating the CRL5 E3 ligase-mediated ubiquitination of NOXA. A subsequent study demonstrated that PRDX1 oligomers, induced by ROS, can bind with CUL5 and the Nedd8-conjugating enzyme UBE2F, thus facilitating their interaction and the transfer of Nedd8 to CUL5. This PRDX1-induced UBE2F-CUL5-dependent degradation of NOXA is critical for maintaining homeostasis under metabolic stress conditions and contributes to etoposide resistance in CRC.

## Results

### NOXA is a highly expressed but short-lived protein in CRC

**Fig. 1:**
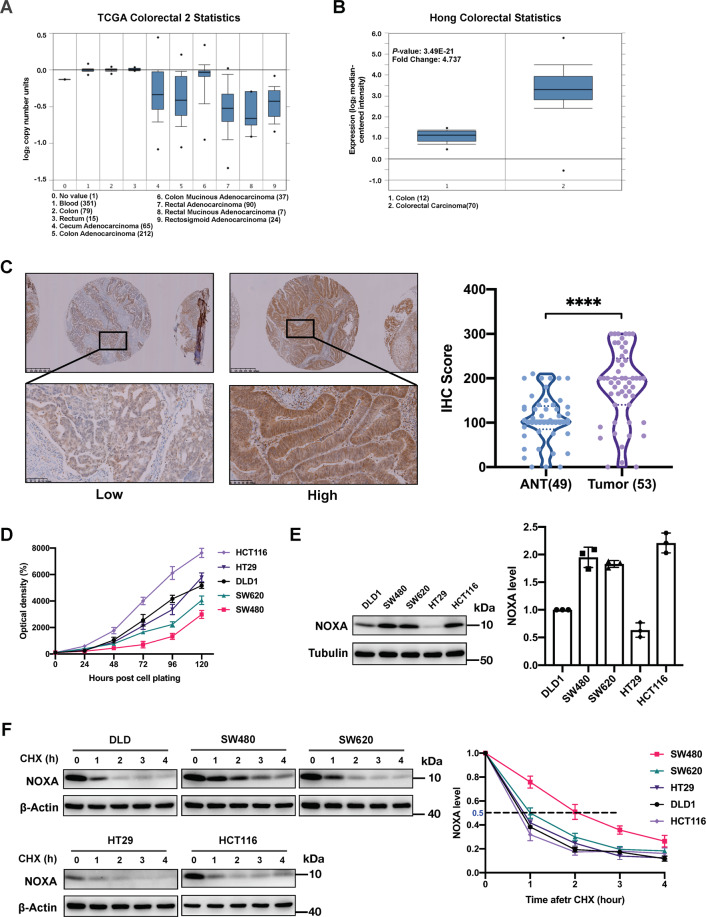
NOXA is a highly expressed but short-lived protein in CRC. **A**, **B** Box plots of NOXA DNA copy numbers (**A**) and mRNA expression levels (**B**) in normal colorectal tissue and colorectal carcinoma tissues in two independent datasets from Oncomine (*n* = number of biologically independent samples). The data sets were taken from TCGA^25^ and Hong^26^. Points: minimum and maximum, whiskers: 10th and 90th percentiles, boxes: 25th and 75th percentiles, lines: medians; unpaired *t* test. **C** Immunohistochemical analysis of NOXA expression levels in ANT (*n* = 49) and CRC tissue (*n* = 53) (****p < 0.0001). The expression level of NOXA was scored from 0 to 300 according to the staining intensity. **D** Comparison of proliferation ability among 5 CRC cell lines (DLD1, SW480, SW620, HT29, and HCT116). Cells were seeded into 96-well plates (2000 cells per well) in triplicate and subjected to a CCK8 assay every 24 h for a duration of 120 h. The mean ± SD of four independent experiments is shown (*p < 0.05). **E** Comparison of NOXA expression in the 5 CRC cell lines by WB analysis. The NOXA level in DLD1 cells on the left was 1, and Tubulin was used as the loading control. The mean ± SD of three independent experiments is shown. **F** Comparison of the NOXA degradation rate in the 5 CRC cell lines by WB analysis. Measurement of NOXA T_1/2_: Cells in 6-well plates were switched to fresh medium (10% FBS) containing CHX (20 μg/mL) and incubated for the indicated time periods before being harvested for WB analysis. The results are representative of three independent experiments, and band densities were quantified using Image Lab software and plotted using Prism 8 software (mean ± SD).

To characterize NOXA from the genome to the protein level in CRC, we surveyed the TCGA dataset of CRC patients in Oncomine. Although there was significant copy number loss of the NOXA gene in CRC tissues compared to normal tissues (Fig. 1A)^25^, the *NOXA* mRNA transcript level was still 2-3-fold higher in CRC tissues than in ANTs (Fig. 1B)^26^. Consistent with these findings, immunohistochemical staining of NOXA demonstrated that CRC tissues indeed exhibited higher NOXA protein levels than did ANTs (Fig. 1C), indicating that translation of NOXA in CRC is not compromised. Unexpectedly, overexpression of NOXA (both mRNA and protein) in CRC did not show a correlation with any clinical parameter analyzed, including overall survival, tumor stage and sex (Fig. S3). Thus, one unknown that needs to be identified is the mechanism by which CRC cells maintain their proliferative ability even with high levels of NOXA, which is a well-known apoptosis inducer.

We further profiled the NOXA protein level in 5 CRC cell lines and analyzed its association with cell proliferation. Although HCT116 cells exhibited the highest proliferation ability among the cell lines tested, they expressed NOXA protein at a level similar to that in SW480 cells, which exhibited much slower proliferation than the other cell lines (Fig. 1D, E), indicating that another factor may account for the disparity. Then, the 5 cell lines were subjected to a cycloheximide (CHX) chase assay to determine the turnover rate of NOXA. As shown in Fig. 1F, NOXA is indeed a short-lived protein in CRC cells, with a half-life ranging from 0.7 h (HCT116) to 2 h (SW480), consistent with a report in lung cancer^20^. More importantly, a strong correlation was observed between the NOXA protein half-life and cell proliferation ability, as cell lines with a more rapid NOXA turnover rate had higher proliferative potential (Fig. 1D, F). During carcinogenesis, the UPS is exploited by cancer cells to accelerate the degradation of tumor suppressor proteins, in one sense reducing their abundance, and in another sense restricting their action time or activity by decreasing their half-life, thereby minimizing the negative effects of tumor suppressors^27,28^. These data indicate that timely degradation of newly synthesized NOXA may be one of the strategies developed by CRC cells to circumvent the risk of high NOXA transcription.

### PRDX1 inhibits NOXA-dependent apoptosis of CRC cells

**Fig. 2:**
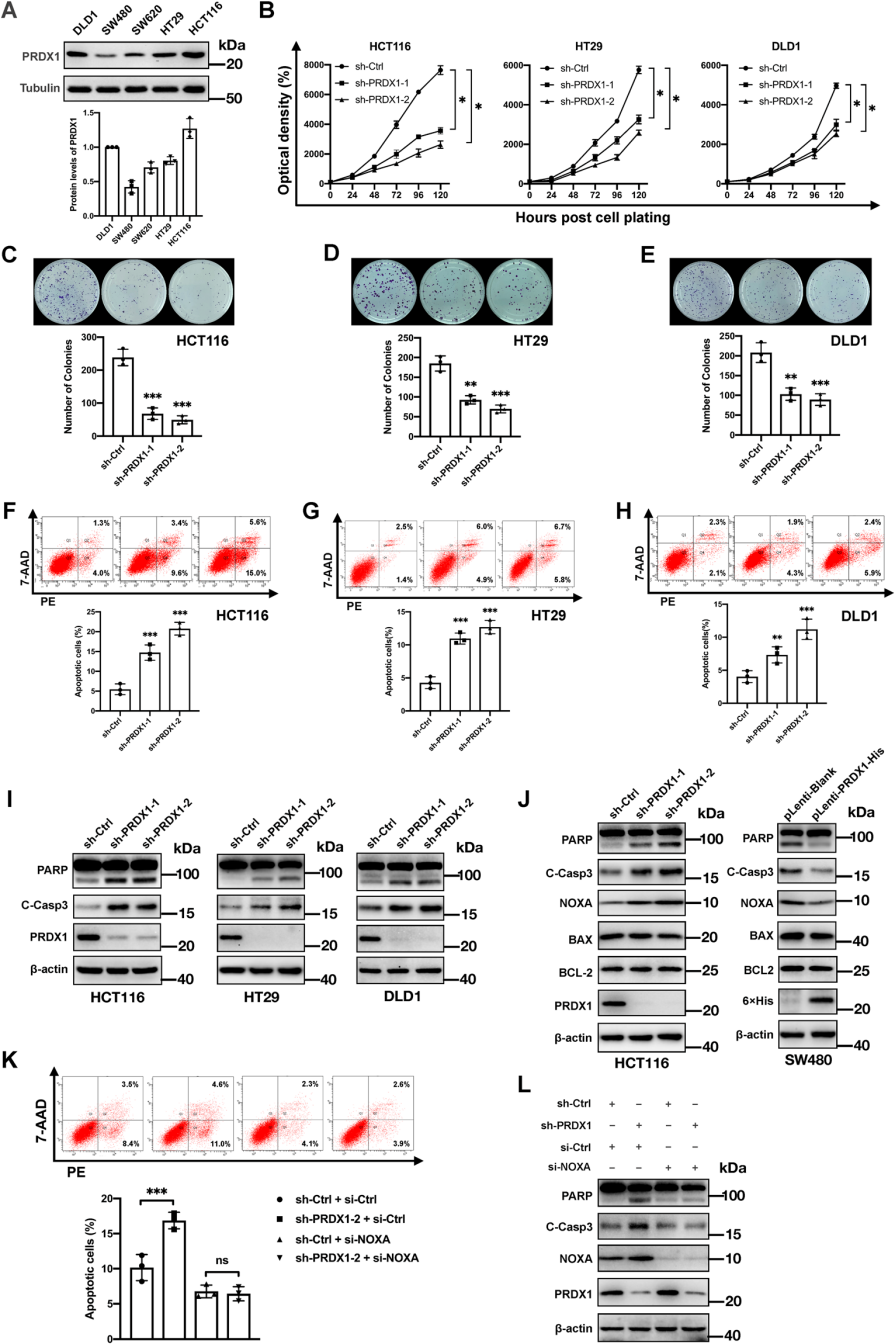
PRDX1 inhibits NOXA-dependent apoptosis of CRC cells. **A** Comparison of PRDX1 expression in different CRC cell lines (DLD1, SW480, SW620, HT29, and HCT116) by WB analysis. The PRDX1 level in DLD1 cells was set as 1, and tubulin was used as the loading control. **B** Cell growth assay: After shRNA-mediated silencing of PRDX1, cells were seeded into 96-well plates (2000 cells per well) in triplicate and subjected to a CCK8 assay every 24 h for a duration of 120 h. The mean ± SD of four independent experiments is shown (*p < 0.05). **C**–**E** Clonogenic survival assay. After shRNA-mediated silencing of PRDX1, HCT116 (**C**), HT29 (**D**), and DLD1 (**E**) cells were seeded into 60-mm dishes (1000 cells per dish). After 2 weeks of incubation at 37 °C, colonies were stained with 0.1% crystal violet and counted. The mean ± SD of three independent experiments is shown (**p < 0.01, ***p < 0.001). **F**–**H** HCT116 (**F**), HT29 (**G**), and DLD1 (**H**) cells stained with 7-AAD and PE were analyzed by flow cytometry to detect apoptosis induced by shRNA-mediated silencing of PRDX1 in CRC cell lines. The mean ± SD of three independent experiments is shown (**p < 0.01, ***p < 0.001). **I** PRDX1 knockdown induced apoptosis in CRC cells: cells infected with lentivirus expressing the indicated shRNAs were subjected to WB analysis with β-actin as the loading control. **J** The expression of NOXA but not BCL2 or BAX was changed upon PRDX1 silencing in HCT116 cells or PRDX1 overexpression in SW480 cells. Cells transduced with the indicated lentiviral vectors were subjected to WB analysis. **K**, **L** Apoptosis caused by knockdown of PRDX1 was suppressed by NOXA silencing. HCT116 cells infected with lentivirus expressing the indicated shRNAs were transfected with si-Ctrl and si-NOXA for 48 h prior to flow cytometry (**K**) and WB analysis (**L**). The NOXA protein and apoptosis markers (cleaved PARP and cleaved caspase-3) were analyzed. The mean ± SD of three independent experiments is shown (***p < 0.001, ns: no significant difference).

The overexpression- and tumor-promoting effects of PRDX1 have been described in numerous types of human cancer^29–31^. Herein, our immunohistochemical staining results demonstrated that PRDX1 is also overexpressed in CRC tissues compared to ANTs (Fig. S4). Although previous reports have shown that PRDX1 expression predicts poor prognosis in CRC^30^, the underlying mechanism by which PRDX1 promotes CRC progression has not been clarified. Among the 5 CRC cell lines used in this study, HCT116, HT29 and DLD1 cells, which have high proliferative potential, expressed higher levels of PRDX1 than the other two cell lines (SW480 and SW620) (Figs. 2A and 1D). We therefore targeted PRDX1 using shRNA-mediated silencing in these 3 cell lines to verify its necessity in CRC. As shown in Fig. 2B, PRDX1 silencing significantly inhibited cell growth in all three cell lines, as measured by a CCK8 assay. Furthermore, clonogenic survival of these three CRC cell lines was significantly decreased upon PRDX1 depletion (Fig. 2C–E). In all 3 cell lines, cell survival was reduced upon PRDX1 silencing via induction of apoptosis, as evidenced by the results of flow cytometric analysis (Fig. 2F–H) as well as the cleavage of caspase 3 and PARP, which play central roles in apoptosis execution (Fig. 2I). Subsequently, we investigated several potential upstream regulators of apoptosis, such as BAX, BCL-2, and NOXA. As shown in Fig. 2J, the protein level of only NOXA apparently increased upon PRDX1 silencing in HCT116 (PRDX1-high) cells. Conversely, overexpression of PRDX1 in SW480 cells (PRDX1-low) significantly reduced the protein level of NOXA without any influence on the protein level of BCL-2 or BAX. To verify whether the increased level of NOXA is responsible for PRDX1 inhibition-induced apoptosis, we performed a rescue experiment and found that simultaneous silencing of PRDX1 and NOXA significantly inhibited the apoptosis caused by PRDX1 silencing; this effect was accompanied by a reduction in the cleavage of caspase 3 and PARP (Fig. 2K, L). These results strongly suggest that PRDX1 inhibits apoptosis in CRC cells by negatively regulating NOXA.

### PRDX1 promotes UPS-mediated degradation of NOXA in CRC cells

**Fig. 3:**
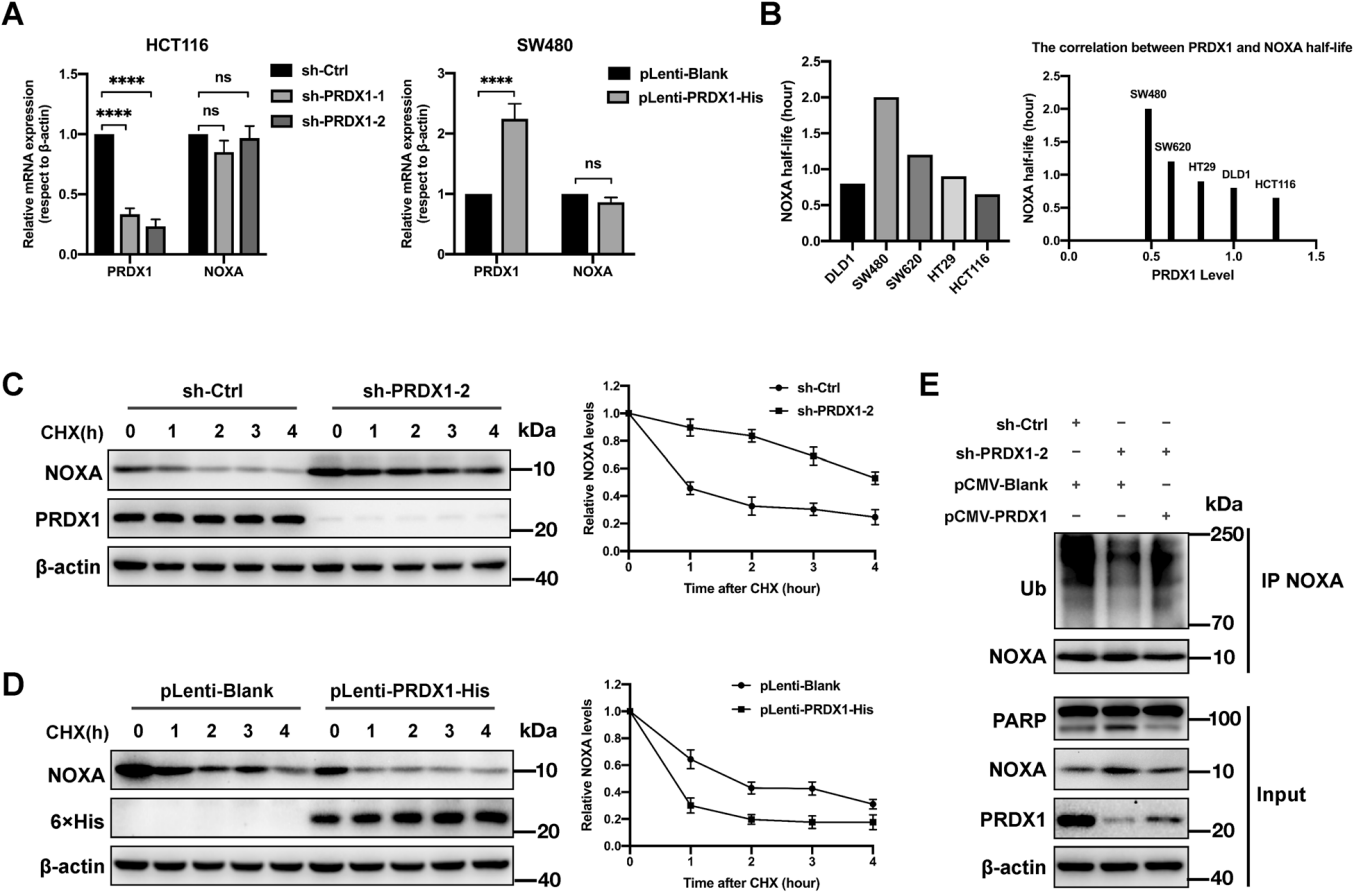
PRDX1 promotes UPS-mediated degradation of NOXA in CRC cells. **A** mRNA levels of PRDX1 and NOXA with respect to those of β-actin in CRC cells after PRDX1 inhibition in HCT116 cells or PRDX1 overexpression in SW480 cells (*n* = 12, mean ± SD; ****p < 0.0001). Cells transduced with the indicated shRNAs or plasmids were subjected to qPCR analysis. **B** Comparison of the NOXA half-life (based on Fig. 1F) and PRDX1 protein level (based on Fig. 2A) in the 5 CRC cell lines. **C**, **D** Measurement of the NOXA degradation rate influenced by PRDX1: HCT116 (**C**) cells were transfected with shRNA to knock down PRDX1, while SW480 (**D**) cells were infected with recombinant lentivirus loaded with pLenti-PRDX1-His (pLenti-Blank as the control) and screened in BSD for 120 h. The obtained cells were switched to fresh medium (10% FBS) containing CHX (20 μg/mL) and incubated for the indicated time periods before being harvested for WB analysis. **E** HCT116 cells infected with lentivirus expressing the indicated shRNAs, were complemented with the indicated plasmids (pCMV-blank, pCMV-PRDX1) and treated with 10 μM MG132 for 10 h before co-IP and WB analyses.

To further investigate the potential mechanism by which PRDX1 downregulates NOXA, we performed qPCR analysis on HCT116 cells transfected with PRDX1-targeting shRNAs and SW480 cells transduced with a PRDX1 overexpression lentiviral vector. In contrast to the results observed in the control groups, both the silencing and overexpression strategies were effective but did not show any significant impact on the transcription of NOXA mRNA (Fig. 3A), implying that the transcription-independent pathway is responsible for PRDX1’s regulatory effect on NOXA. When assessing correlation between the level of PRDX1 and the turnover rate of NOXA in the 5 CRC cell lines, we found a negative correlation between the PRDX1 protein level and the NOXA protein half-life: the higher the protein level of PRDX1 was, the lower the NOXA protein half-life (Fig. 3B). Indeed, knockdown of PRDX1 in HCT116 cells greatly decelerated the turnover of NOXA, extending its half-life from 1.5 h to 4 h (Fig. 3C). Moreover, PRDX1 overexpression in SW480 cells apparently accelerated the degradation of NOXA (Fig. 3D). To validate this finding, we tested whether PRDX1 impacts the ubiquitination of NOXA. As shown in Fig. 3E, silencing of PRDX1 in HCT116 cells significantly reduced the ubiquitin conjugation to NOXA, and further transient expression of PRDX1 in these PRDX1-silenced HCT116 cells rescued the ubiquitination of NOXA. Collectively, the above data provide convincing evidence that PRDX1 promotes ubiquitin-mediated degradation of NOXA in CRC cells.

### PRDX1 specifically promotes CUL5 neddylation by connecting it to UBE2F

**Fig. 4:**
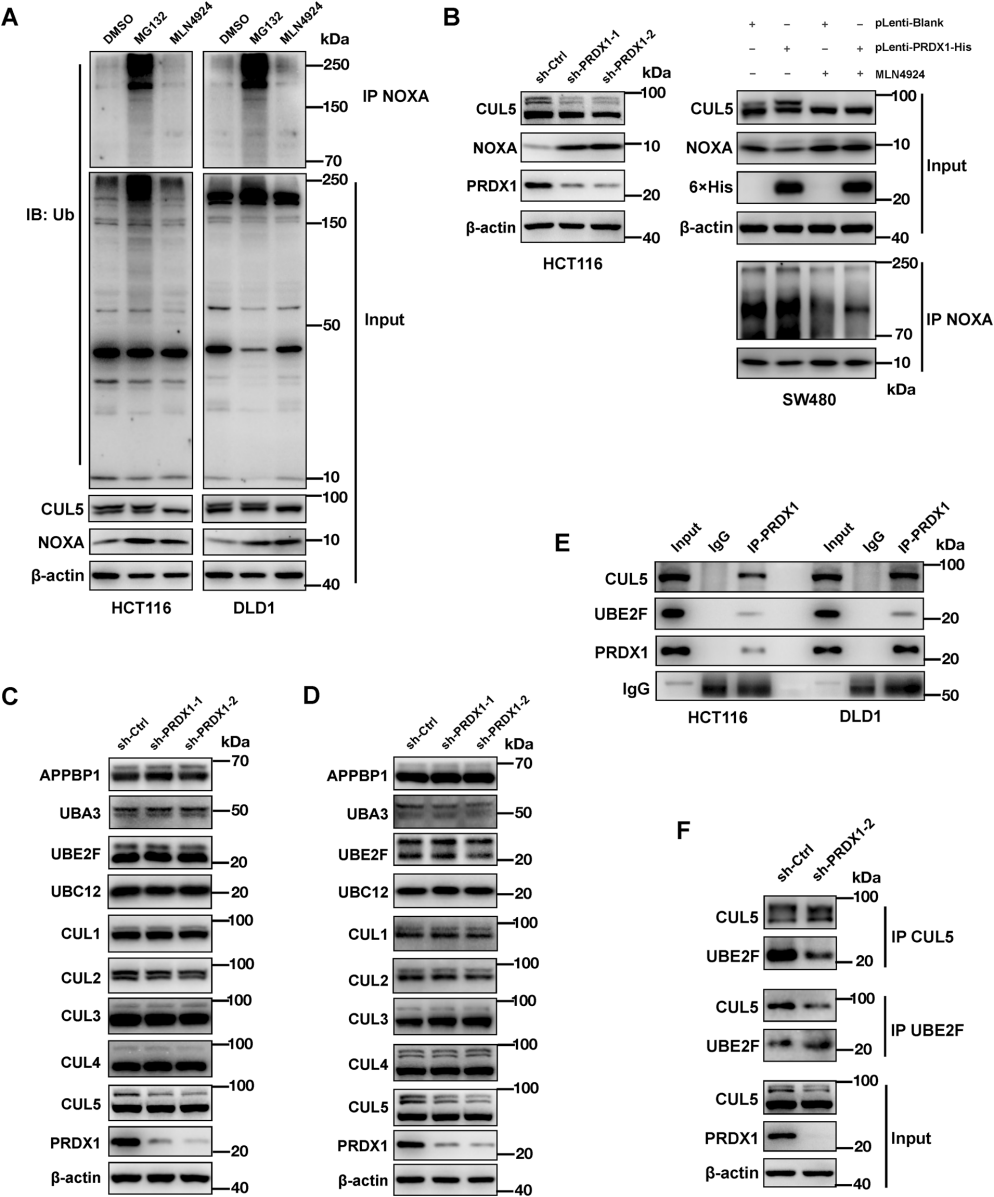
PRDX1 specifically promotes CUL5 neddylation by connecting it to UBE2F. **A** HCT116 cells were treated with DMSO (as a control), MG132 (10 μM), or MLN4924 (1 μM) for 10 h prior to co-IP and WB analyses. **B** In HCT116 cells, PRDX1 inhibition led to a decrease in CUL5 neddylation and an increase in NOXA expression, while the opposite phenomenon occurred in PRDX1-overexpressing SW480 cells. These effects of PRDX1 overexpression on CUL5 neddylation and NOXA ubiquitination in SW480 cells were abolished when the same two groups of cells were treated with MLN4924: SW480 cells transduced with pLenti plasmids were treated with 1 μM MLN4924 and/or 10 μM MG132 for 10 h before co-IP and WB analyses. **C**, **D** WB analysis of the changes in E1 activating enzymes (APPBP1-UBA3), E2 conjugating enzymes (UBE2F and UBC12) and CUL family proteins (CUL1-5). HCT116 (**C**) and DLD1 (**D**) cells were infected with lentivirus expressing the indicated shRNAs before being harvested for WB analysis. **E** PRDX1, UBE2F, and CUL5 bind to each other. HCT116 and DLD cell lysates were immunoprecipitated using an anti-PRDX1 or IgG control antibody and subsequently with Protein A + G Agarose prior to WB analysis to detect endogenous proteins as indicated. A 10% aliquot of each extract was used as input. **F** HCT116 cells were transfected with sh-PRDX1 before the lysates were immunoprecipitated using an anti-UBE2F or anti-CUL5 antibody and subsequently with Protein A + G Agarose prior to WB analysis to further determine the relationship of the three indicated proteins.

Among the potential neddylation substrates, the cullin family members (CUL-1, -2, -3, -4A, -4B, and -5) are well defined^32^. The neddylation of CUL5 activates CRL5 to ubiquitinate NOXA via a novel K11 linkage for targeted proteasomal degradation^16^. Here, we determined that in CRC cells, the degradation of NOXA is also totally dependent on the neddylation pathway. This conclusion was based on the finding that treatment with MG132 (a proteasome inhibitor)^27,33^ or MLN4924 (a NEDD8-activating enzyme inhibitor)^34^ obviously increased the NOXA protein level, but only MG132 led to accumulation of ubiquitinated NOXA, while MLN4924 completely abolished CUL5 neddylation (Fig. 4A).

As PRDX1 promoted the degradation of NOXA (Fig. 3), we determined whether it affects the neddylation of CUL5, which is a direct activator of the CRL5 E3 ligase. Silencing of PRDX1 in HCT116 cells significantly reduced the neddylation of CUL5; consistent with this pattern, increased neddylation of CUL5, as well as an increase in NOXA ubiquitination and a decrease in the NOXA protein level, were observed in SW480 cells overexpressing PRDX1, and these effects of PRDX1 overexpression on CUL5 neddylation and NOXA ubiquitination were abolished when the same two groups of cells were treated with MLN4924 (Fig. 4B). This finding indicates that PRDX1 promotes NOXA degradation by enhancing CUL5 neddylation. To date, one heterodimeric Nedd8 E1 activating enzyme (APPBP1–UBA3) and two NEDD8 E2 conjugating enzymes (UBE2F and UBEC12) have been found to act cooperatively in the neddylation reaction^35^. However, knockdown of PRDX1 in HCT116 and DLD1 cells neither changed the protein levels of these upstream E1 or E2 enzymes nor modified their enzymatic activity, as demonstrated by the similar levels of Nedd8 conjugation to these enzymes and to the CUL1-4 proteins (Fig. 4C, D). Thus, the role of PRDX1 in the neddylation pathway is specific to CUL5.

Known the Nedd8 molecule is conjugated to CUL5 specifically from UBE2F^32^, to verify our hypothesis that PRDX1 may impact the interaction between CUL5 and UBE2F, which is essential for CUL5 neddylation^16^, we performed co-IP assays in HCT116 and DLD1 cell lines. As shown in Fig. 4E, both CUL5 and UBE2F were present in the anti-PRDX1 immunoprecipitate, indicating the potential interaction of these three proteins. Interestingly, silencing of PRDX1 significantly reduced the protein level of UBE2F in the anti-CUL5 immunoprecipitate, a finding that was reproduced when an antibody against UBE2F was used for co-IP assays (Fig. 4F). In summary, PRDX1 can function as a bridge between UBE2F and CUL5, an interaction that is required for CUL5 neddylation and subsequent NOXA ubiquitination.

### Oligomerization of PRDX1 is required for CUL5 neddylation and NOXA degradation

**Fig. 5:**
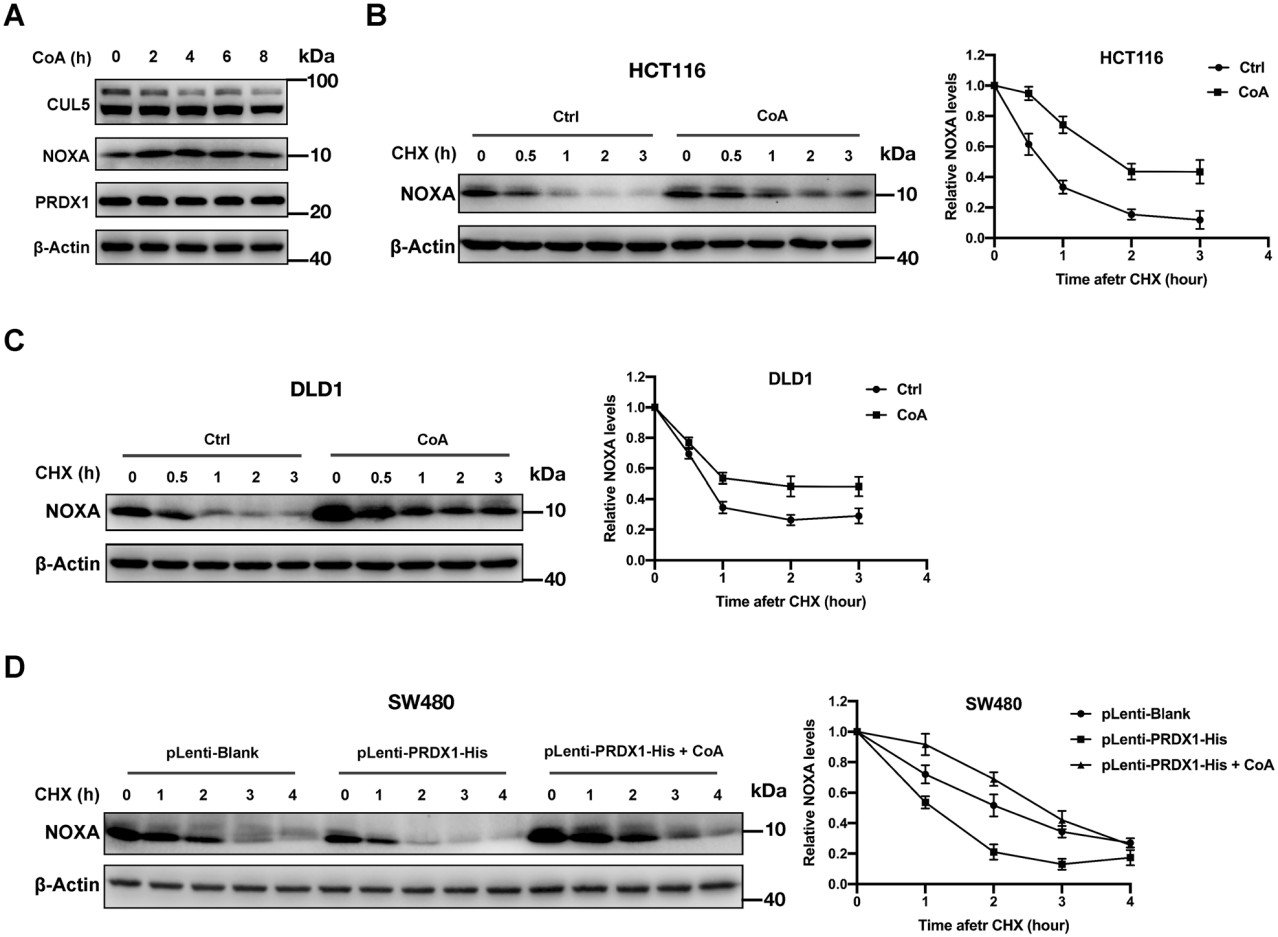
Oligomerization of PRDX1 is required for CUL5 neddylation and NOXA degradation. **A** WB analysis of PRDX1, CUL5 neddylation and NOXA protein levels in HCT116 cells treated with CoA (10 μM) for 0~8 h. HCT116 cells treated with CoA for a fixed time were lysed with RIPA buffer before WB analyses. **B**–**D** Analysis of the influence of the PRDX1 oligomerization inhibitor CoA on the degradation rate of NOXA: HCT116 (**B**), DLD1 (**C**) and transduced SW480 (**D**) cells in 6-well plates were pretreated with CoA (10 μM) for 2 h prior to CHX treatment for a fixed duration before being lysed for WB analysis. The results are representative of three independent experiments, and band densities were quantified using Image Lab software and plotted using Prism 8 software (mean ± SD).

To identify the form of PRDX1 that potentially interacts with CUL5 and UBE2F, we studied whether oligomerization of PRDX1 is required for CUL5 neddylation by treating CRC cells with Conoidin A (CoA, a PRDX1 inhibitor that covalently binds to the catalytic cysteine) to inhibit the oligomerization of PRDX1 (ref. ^36,37^). As shown in Fig. 5A, the total protein level of PRDX1 was unaffected by CoA treatment, and the increase in the protein level of NOXA upon CoA treatment was accompanied by a decrease in the neddylation of CUL5 in a time-dependent manner. In addition, inhibition of PRDX1 oligomers with CoA significantly extended the NOXA protein half-life in HCT116 cells (Fig. 5B) and DLD1 cells (Fig. 5C) compared to that in their control groups. Although the over-expression of PRDX1 significantly reduced the protein half-life of NOXA in SW480 cells, this effect was completely abolished by addition of CoA (Fig. 5D). Taken together, these results demonstrate that oligomerization of PRDX1 is required for CUL5 neddylation and subsequent NOXA degradation in CRC cells.

### ROS reduce the NOXA protein half-life by inducing PRDX1 oligomerization

**Fig. 6:**
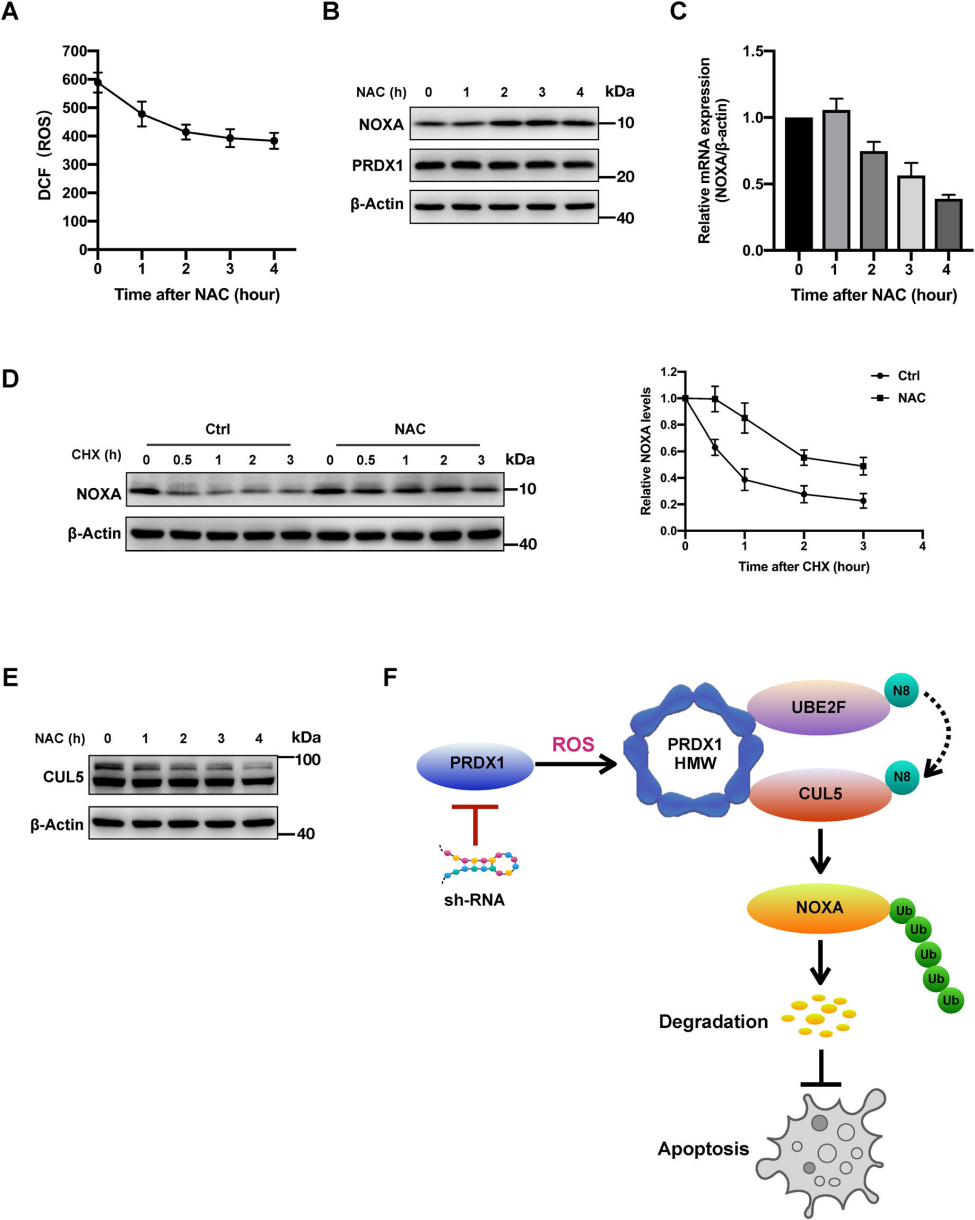
ROS reduce the NOXA protein half-life by inducing PRDX1 oligomerization. **A** NAC induced a reduction in total ROS levels in HCT116 cells: HCT116 cells in a 6-well plate were treated with 5 mM NAC for 0–4 h and were then analyzed by flow cytometry to measure the total intracellular ROS level. The mean ± SD values of three experiments were plotted using Prism 8 software. **B** Under NAC treatment, the protein level of NOXA apparently increased, while the total protein level of PRDX1 was unaffected: HCT116 cells in 6-well plates were treated with 5 mM NAC for 0–4 h and were then lysed with RIPA before WB analysis. **C** NAC treatment reduced NOXA transcription: mRNA levels of NOXA with respect to those of β-actin in HCT116 cells after treatment with 5 mM NAC for 0–4 h. *n* = 12, mean ± SD, plotted using Prism 8 software. **D** Analysis of the influence of NAC on the degradation rate of NOXA: HCT116 cells in 6-well plates were pretreated with NAC (5 mM) for 1 h prior to CHX treatment for a fixed duration before being lysed for WB analysis. The results are representative of three independent experiments, and band densities were quantified using Image Lab software and plotted using Prism 8 software (mean ± SD). **E** Analysis of the influence of NAC on CUL5 neddylation: HCT116 cells in 6-well plates were pretreated with NAC (5 mM) for 0–4 h before being lysed for WB analysis. **F** A working model: PRDX1 protects CRC cells from NOXA-dependent apoptosis by promoting NOXA degradation through enhancing CUL5 neddylation.

A key player in oxidation resistance, PRDX1 itself is hyperoxidized to form oligomers after scavenging high level of ROS^19–21^. Our above data show that PRDX1 oligomers bind both UBE2F and CUL5 and are required for CUL5 neddylation in CRC cells. This finding suggests that the cellular ROS level may be an upstream regulator of PRDX1 in its control of CUL5 neddylation and subsequent NOXA degradation. To verify this hypothesis, we treated HCT116 cells with N-acetyl-L-cysteine (NAC, a commonly used ROS scavenger) to reduce cellular ROS levels^38,39^. As expected, along with the reduction in ROS levels upon NAC treatment (Fig. 6A), NOXA protein levels were significantly elevated 2 h after NAC treatment, although the total protein level of PRDX1 was unaffected (Fig. 6B); this effect was not due to an increase in NOXA transcription, as ROS scavenging negatively impacted the mRNA level of NOXA (Fig. 6C). We then treated CRC cells simultaneously with NAC and CHX to test the impact of ROS inhibition on the NOXA protein half-life. As shown in Fig. 6D, in contrast to the control treatment, NAC treatment significantly extended the endogenous NOXA protein half-life to more than 2 h in HCT116 cells, indicating that ROS promote the degradation of NOXA post-transcriptionally by facilitating PRDX1 oligomerization. Consistent with this result, ROS scavenging by NAC also led to a significantly reduced level of CUL5 neddylation (Fig. 6E). Collectively, these results reveal an unexpected role of intracellular ROS in enhancing CUL5 neddylation and reducing the NOXA protein half-life (Fig. 6F).

### PRDX1 protects CRC cells against etoposide-induced apoptosis by inducing the CUL5-NOXA pathway

The above data demonstrate that to maintain their high proliferative capacity, CRC cells rapidly degrade newly synthesized NOXA by enhancing the activity of its ubiquitin E3 ligase CRL5, which is at least partially controlled by PRDX1 through linking UBE2F with CUL5. Then, the following question is raised: under stress conditions when NOXA is easily induced, especially chemotherapy-induced DNA damage, does PRDX1-regulated CUL5 neddylation play roles in drug resistance? To answer this question, we treated CRC cells with etoposide, a well-known anticancer drug that induces DNA damage^40–42^.

**Fig. 7:**
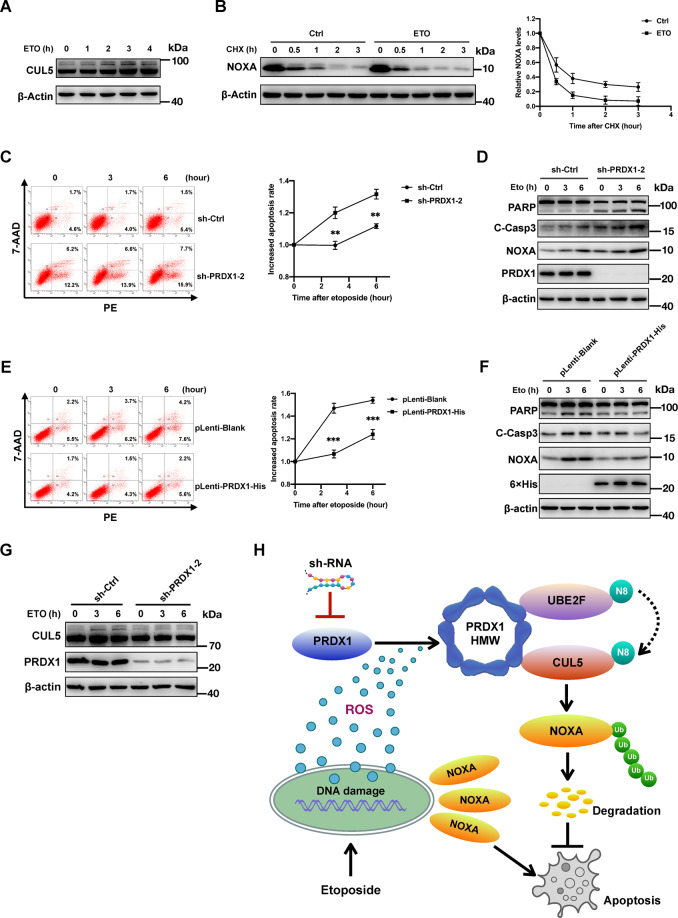
PRDX1 protects CRC cells against etoposide-induced apoptosis by inducing the CUL5-NOXA pathway. **A** Analysis of the influence of etoposide on CUL5 neddylation: HCT116 cells in 6-well plates were pretreated with etoposide (50 mM) for 0–4 h before being lysed for WB analysis. **B** Analysis of the influence of etoposide on the NOXA degradation rate: HCT116 cells in 6-well plates were pretreated with etoposide (50 mM) for 1 h prior to CHX treatment for a fixed duration before being lysed for WB analysis. The results are representative of three independent experiments, and band densities were quantified using Image Lab software and plotted using Prism 8 software (mean ± SD). **C**, **D** Inhibition of PRDX1 increased the sensitivity of HCT116 cells to etoposide-induced apoptosis: HCT116 cells infected with lentivirus expressing the indicated shRNAs were treated with 50 mM etoposide for 0–6 h and were then analyzed by flow cytometry (**C**) to detect apoptosis (mean ± SD; **p < 0.01) or harvested for WB analysis (**D**) of NOXA and apoptosis marker proteins (cleaved PARP and cleaved c-Casp3). The results are representative of three independent experiments. **E**, **F** Overexpression of PRDX1 protected SW480 cells against etoposide-induced apoptosis: SW480 cells infected with the indicated pLenti-DNA recombinant lentiviruses were treated with 50 mM etoposide for 0–6 h and were then analyzed by flow cytometry (**E**) to detect apoptosis (mean ± SD; ***p < 0.001) or harvested for WB analysis (**F**) of NOXA and apoptosis marker proteins (cleaved PARP and cleaved c-Casp3). The results are representative of three independent experiments. **G** The increase in CUL5 neddylation induced by etoposide was PRDX1-dependent: HCT116 cells infected with lentivirus expressing the indicated shRNAs were treated with 50 mM etoposide for 0–6 h and were then harvested for WB analysis of CUL5 neddylation. **H** A working model: PRDX1 protects CRC cells against etoposide-induced apoptosis by inducing the UBE2F-CUL5-NOXA pathway.

As a DNA topoisomerase II inhibitor, etoposide generally increases p53 expression and NOXA transcription^41^, which was also evident in our study (Fig. S5). Interestingly, upon etoposide treatment, CUL5 neddylation in HCT116 cells was increased in a time-dependent manner (Fig. 7A), accompanied by a reduction in the NOXA protein half-life compared to that in the control group (Fig. 7B). When PRDX1 was silenced in HCT116 cells and these cells were treated with etoposide, knockdown of PRDX1 significantly increased the percentage of apoptotic cells, based on flow cytometric analysis, and led to an increase in the NOXA protein level and cleavage of caspase-3 and PARP (Fig. 7C, D). This result indicates that the absence of PRDX1 increases the sensitivity of HCT116 cells to etoposide. Consistent with this, overexpression of PRDX1 in SW480 cells noticeably reduced apoptosis and the levels of the associated proteins (NOXA, cleaved caspase-3 and cleaved PARP) in response to etoposide (Fig. 7E, F). Thus, these results confirm that PRDX1 is required for the resistance of CRC cells to etoposide-induced apoptosis: although DNA damage caused by etoposide increases the transcription of NOXA mRNA, CRC cells induce the neddylation pathway to accelerate NOXA degradation, thereby minimizing its apoptosis-inducing effect.

Subsequently, we found that in HCT116 cells with PRDX1 silencing, the time-dependent increase in CUL5 neddylation upon etoposide treatment was abolished (Fig. 7G). Consistent with the role of ROS in PRDX1 oligomerization, which is critical for CUL5 neddylation, etoposide treatment also transiently increased the cellular level of ROS, which peaked at ~1 h post treatment (Fig. S6). Taken together, our data clearly demonstrate that PRDX1-mediated CUL5 neddylation and subsequent NOXA degradation must be one of the strategies by which CRC cells counteract the toxic effect of chemotherapeutics (Fig. 7H).

## Discussion

Among all BH3-only proteins identified to date, NOXA appears to be crucial in fine-tuning cell death decisions by targeting the prosurvival molecule MCL1 for degradation^12,13^. In our study, we characterized NOXA in CRC and found that CRC tissues expressed significantly higher levels of NOXA mRNA and protein than ANTs despite the evident *NOXA* copy number loss in CRC tissues. This pattern is highly possible, since NOXA is readily inducible by several transcription factors (p53, p73, HIF1a, E2F1, c-MYC, ATF3/4, etc.) under stress conditions, such as hypoxia and exposure to chemotherapeutics, which are very common in cancer tissues^13,14^. An intriguing question is the following: since neither the transcription nor translation of this proapoptotic protein is compromised in CRC, how do CRC cells circumvent this limitation to maintain their high proliferative potential? After screening the turnover rate of NOXA in 5 distinct CRC cell lines, we observed that NOXA is a short-lived protein with a half-life of less than 2 h, as also reported in leukemia and lung cancer^15,16^. This observation indicates that although the expression of NOXA is highly induced in CRC, its rapid degradation not only restricts its action time but also prevents its overaccumulation. Accordingly, CRC cell lines with a shorter NOXA protein half-life have a stronger proliferative ability, which may partially answer the above question. This also indicates that ubiquitination pathway associated with NOXA degradation may be hijacked by CRC cells.

NOXA mediated apoptosis of cancer cells that is consequent to oxidative stress has been extensively reported in the past decades, it has been made clear that how NOXA was transcriptionally regulated under oxidative stress conditions which is common for cancer cells with high metabolic activity^43,44^. However, to date, whether ubiquitination pathway associated with NOXA degradation was simultaneously altered under oxidative stress conditions and how is this coordinated have been rarely reported. PRDX1, known as one member of peroxiredoxin family, has been demonstrated to have tumor-promoting^29–31^ and apoptosis-inhibiting^45–47^ role in several types of cancer. In this research, we found that PRDX1 staining was significantly higher in CRC tissues than in ANTs and that PRDX1 can inhibit cancer cell apoptosis by decreasing NOXA levels. Mechanistically, this effect of PRDX1 was found to be due to its role in promoting NOXA degradation by ubiquitination, which strongly suggested that PRDX1 may activate CRL5 (E3 ligase specific for NOXA ubiquitination) via an unknown mechanism. Profiling potential targets of PRDX1 in the neddylation pathway revealed that PRDX1 did not influence the expression levels or activities of any Nedd8-E1 and -E2 enzymes tested except CUL5 neddylation. This is the first line of evidence indicating that PRDX1 specifically promotes CUL5 neddylation, which is a direct activator of CRL5.

In view of its native antioxidant function, PRDX1 may be the upstream regulator that transduces oxidative stress signal to CUL5 neddylation pathway that controls NOXA ubiquitination. However, CRC cells lacking PRDX1 still have considerable basal levels of neddylated CUL5 protein, implying that PRDX1 probably acts as an “enhancer” rather than a “switch” of CRL5 activity. Alternatively, other unknown factors may also contribute to this process. It is also worth determining whether other members of the PRDX family perform similar functions as PRDX1 in promoting CUL5 neddylation and NOXA degradation.

After undergoing hyperoxidation, PRDX1 forms HMW complex and thus exhibits chaperone activity^20,21^. PRDX1 oligomers have been shown to mediate cell signaling events by regulating the activity of their binding partners, such as JNK, c-Abl kinase and the phosphatase PTEN^48^. As more binding partners are identified, the biological versatility of PRDX1 is being recognized. Here, we found that in CRC cells, PRDX1 binds with both UBE2F and CUL5 to form a complex. This PRDX1-UBE2F-CUL5 trimer may provide a platform for connecting UBE2F and CUL5, because silencing PRDX1 expression significantly reduced the abundance of CUL5 in UBE2F immunoprecipitates and vice versa. That effect was accompanied by reduced CUL5 neddylation. In addition, modifying PRDX1 expression in CRC cells did not influence either the mRNA or protein expression of SAG (RNF7), a well-known adaptor for UBE2F and CUL5^49^; moreover, SAG was also present in the anti-PRDX1 immunoprecipitate (Figs. S7 and S8). Accordingly, the connection of UBE2F with CUL5 through PRDX1 is dependent on their direct interaction with PRDX1, not due to an influence of PRDX1 on SAG expression. The importance of PRDX1 oligomers in this process was emphasized by the result that inhibition of PRDX1 oligomerization by CoA markedly reduced CUL5 neddylation and extended the NOXA protein half-life. Thus, it is most likely that through the formation of this trimeric complex, PRDX1 facilitates the transfer of Nedd8 from UBE2F to CUL5. However, we still cannot exclude the possibility that PRDX1 also functions as an intermediate or E3 ligase in the unidirectional transfer of Nedd8 from UBE2F to CUL5. In future studies, identifying the interaction motifs in these three proteins and analyzing their crystal structures will help to clarify this issue.

ROS are the direct driving force for PRDX1 hyperoxidation, and elevated level of ROS is a common hallmark of cancer^50–52^. When we used NAC to scavenge intracellular ROS in CRC cells, the amount of neddylated CUL5 protein was significantly decreased, and the NOXA protein half-life was significantly extended. Unexpectedly, scavenging intracellular ROS with NAC also led to reduced NOXA mRNA transcription via an unknown mechanism. This study revealed, for the first time, the dual functional roles of ROS in regulating NOXA expression. On the one hand, ROS favor *NOXA* mRNA transcription, and on the other hand, ROS accelerate NOXA protein degradation. This may be one of the mechanisms by which CRC cells maintain homeostasis under conditions of high metabolic activity.

In addition, siRNA-mediated silencing of *NOXA* expression in CRC cells did not show any significant impact on either intracellular ROS levels or the protein levels of PRDX1 and neddylated CUL5 (Fig. S9). This finding indicates that the transcriptional and post-transcriptional regulatory pathways controlling the NOXA abundance are completely independent of each other and that the stress caused by increased *NOXA* transcription is not the driving force activating the pathway for its PRDX1-mediated degradation. Furthermore, we found that *PRDX1* silencing abolished the increase in the level of neddylated CUL5 protein induced by etoposide, increasing the sensitivity of CRC cells to etoposide treatment (Fig. 7). These results strongly suggest that PRDX1-reinforced CUL5 neddylation also contributes to the resistance of CRC cells to etoposide. Based on these findings, PRDX1 could be a therapeutic target for CRC.

## Materials and methods

### Tissue microarray (TMA) and immunohistochemistry (IHC)

The human CRC tissue and adjacent normal tissue (ANT) microarrays were purchased from Taibosi Biotechnology Co., Ltd. (Xian, China), and the study was approved by the Human Research Ethics Committee of Sir Run Run Shaw Hospital (Hangzhou, China) and conducted according to the principles of the Declaration of Helsinki. Immunohistochemical analysis was performed to study NOXA and PRDX1 protein expression levels: slides were deparaffinized in xylene, rehydrated in a graded alcohol series, and immersed in 1% hydrogen peroxide for 10 min to quench endogenous peroxidase activity. Antigen retrieval was accomplished by incubation in a pressure cooker for 2.5 min in 0.01 M citrate buffer (pH = 6.0). Then, slides were incubated with the primary antibody (NOXA, 1:200, #13654, Abcam, CB, UK; PRDX1, 1:500, #109498, Abcam) for 40 min in a humidified chamber at room temperature. Specimens were stained with 3,3-diaminobenzidine (DAB; K5007, Dako) after being incubated with the secondary antibody (HRP Anti-Rabbit/Mouse, Dako) for 30 min. Finally, sections were counterstained with hematoxylin, dehydrated and mounted. Protein expression levels were evaluated under a microscope (Eclipse 8i, Nikon, Japan), and semiquantitative analysis of the staining intensity scores was performed as previously described^53^.

### Cell culture

A human renal epithelial cell line (293T) and CRC cell lines (DLD-1, SW480, SW620, HT29, and HCT116) were obtained from the Chinese Academy of Sciences Cell Bank (Shanghai, China) between 2013 and 2020 and were recently authenticated by Shanghai Biowing Biotechnology (Shanghai, China). 293T and DLD1 cells were cultured in Dulbecco’s modified Eagle’s medium (DMEM, Gibco) supplemented with 1% penicillin-streptomycin (P/S, Gibco) and 10% fetal bovine serum (FBS, Gibco). SW480, SW620, HT29, and HCT116 cells were maintained in RPMI-1640 medium (Gibco, NY, USA) supplemented with 1% P/S and 10% FBS. All cells were cultured in an incubator with 5% CO_2_ at 37 °C. All cell lines were tested to confirm the absence of mycoplasma contamination.

### shRNAs, siRNAs, plasmids, and transfection

The shRNAs for PRDX1 (LV-PRDX1-RNAi-8774-1/2) and the negative control shRNA (CON077) were designed and synthesized by Shanghai Genechem (Shanghai, China), and transfection was performed using the provided reagent according to the manufacturer’s instructions. The siRNAs targeting NOXA (hPMAIP1siRNA-1/2/3) and negative control (siRNA N-CTL) were supplied by Sunyabio (Hangzhou, China). Plasmid-CMV6-PRDX1 was constructed by inserting the PRDX1 coding sequence (CDS) into the linearized pCMV6/vector (OriGene Technologies, Beijing, China) using a ClonExpress II One Step Cloning Kit (Vazyme, Jiangsu, China). Transfection of siRNAs and plasmids was carried out using a Lipofectamine 3000 Transfection Kit (Invitrogen, NY, USA) following the manufacturer’s instructions.

### Cloning of PRDX1-His, lentivirus production and infection

The PCR amplification primers used for PRDX1-His cloning are listed in Table S1. The 618 bp DNA fragment, which included the PRDX1 CDS and a 6 × His tag site, was integrated into the pLenti-Basic vector, and all constructs were verified by DNA sequencing. For lentivirus production, 293T cells were cotransfected with pLenti-PRDX1-His and the packaging plasmids pMD2.G and psPAX2 using Lipofectamine 3000. The culture medium was removed after 6–8 h and replaced with fresh DMEM supplemented with 10% FBS. Lentiviruses were collected 48 h after fresh medium replacement and filtered through a 0.45-μm membrane (Millex-HV, Millipore, MA, USA). To achieve stable overexpression of PRDX1, SW480 cells seeded in 30-mm dishes were infected with pLenti-PRDX1-His or pLenti-Blank lentiviral constructs and selected with blasticidin (10 μg/mL).

### Cell viability assay

For the cell proliferation assay, HCT116, DLD1, and HT29 cells were seeded into 96-well plates (2000 cells per well) in triplicate and cultured for 24, 48, 72, 96, and 120 h. A cell proliferation assay was then conducted with a Cell Counting Kit-8 (CCK8, Dojindo Laboratories, Tokyo), according to the instructions of the manufacturer.

For the clonogenic survival assay, HCT116, DLD1 and HT29 cells were seeded into 60-mm dishes (1000 cells per dish) in triplicate and incubated at 37 °C for 2 weeks. The colonies were fixed with methanol, stained with 0.1% crystal violet staining solution, and counted.

### Quantitative real-time polymerase chain reaction (qPCR)

Total RNA from cells was extracted using TRIzol Reagent (Invitrogen, USA) following the manufacturer’s instructions. mRNA levels were analyzed using a HiFiScript cDNA Synthesis Kit (CWBio, China) and a NovoStart SYBR qPCR SuperMix Plus Kit (Novoprotein, China). All expression data were normalized to β-Actin. Relative mRNA expression levels were calculated using the 2^−ΔΔCt^ method. The primers used for qPCR are listed in Table S1.

### Western blot (WB) analysis and coimmunoprecipitation (co-IP) assay

WB analysis was performed as previously described^53^. Cells were lysed in radioimmunoprecipitation assay (RIPA) buffer (P0013B, Beyotime Biotechnology, Shanghai, China) before WB analysis. For co-IP, cells were lysed in mild RIPA lysis buffer (P0013D, Beyotime). The supernatant was first incubated with an antibody overnight, subsequently incubated with Protein A + G Agarose (P2012, Beyotime) at 4 °C for 4 h, and then washed 3 times with lysis buffer. Immunocomplexes were subjected to sodium dodecyl sulfate-polyacrylamide gel electrophoresis (SDS-PAGE) and WB analysis. The antibodies used for WB analysis and co-IP assays are listed in Table S2.

### Apoptosis analysis and ROS measurement by flow cytometry

For apoptosis analysis, the indicated cells were treated as described in 6-well plates, and apoptosis of floating and attached cells was detected with an Annexin V-PE/7-AAD Apoptosis Detection Kit (BD, CA, USA). For measurement of total ROS, the collected attached cells were analyzed with a DCFH-DA Reactive Oxygen Species Assay Kit (Meilunbio, Dalian, China) according to the instructions of the manufacturer. These assays were conducted and analyzed with a BD LSRFortessa^TM^ flow cytometer and the included software.

### NOXA half-life determination

For determination of the NOXA half-life in different CRC cell lines, cells were treated with 20 μg/mL CHX (#28132, MedChemExpress, NJ, USA) for different periods of time (0, 1, 2, 3, and 4 h), and antibodies against endogenous NOXA (β-actin was used as the loading control) were then used for WB analysis. For PRDX1 silencing and overexpression experiments, after lentiviral vector transduction and generation of stable cell lines, cells were treated with CHX (20 μg/mL) for a fixed duration and were then lysed for WB analysis. For CoA, NAC and etoposide experiments, after pretreatment with CoA (C4293, APExBIO, Houston, USA), NAC (MB1735, Meilunbio) or etoposide (#19935, MedChemExpress) for the established times, cells were treated with CHX and lysed for WB analysis. The relative levels of NOXA were quantified photometrically using Image Lab image processing software.

### Quantification and statistical analysis

Each experiment was performed at least three times to obtain data for three biological replicates. For WB and IP experiments, the most representative results of at least three independent experiments with similar results are shown. The quantified points and connecting lines show the average of the indicated independent experiments, and *p* values were obtained by two-way ANOVA with multiple comparisons, with no separate adjustment for multiple comparisons. Statistical analyses were performed using GraphPad Prism 8 (GraphPad Software Inc.), unless otherwise indicated. Data are shown as the mean ± SD or SEM values, as indicated (*p < 0.05, **p < 0.01, ***p < 0.001, ****p < 0.0001).

## Conclusions

Despite its relative overexpression in CRC tissues, NOXA is a short-lived protein whose half-life is at least partially controlled by PRDX1. PRDX1 specifically potentiates CUL5 neddylation, which is a critical activator of the CRL5 E3 ligase-mediated ubiquitination of NOXA. This effect occurs because PRDX1 oligomers, whose formation is induced by ROS, can form a complex with CUL5 and the Nedd8-conjugating enzyme UBE2F, which possibly facilitates the transfer of Nedd8 to CUL5 (Fig. 7H). Collectively, these results support the tumor-promoting effect of ROS and PRDX1 and reveal one of the pathways by which CRC cells maintain homeostasis under metabolic stress.

## Supplementary information

Supplementary 1

Supplementary 2

Supplementary 3

Supplementary 4

Supplementary 5

Supplementary 6

Supplementary 7

Supplementary 8

Supplementary 9
