# Correction: Cullin-5 neddylation-mediated NOXA degradation is enhanced by PRDX1 oligomers in colorectal cancer

**DOI:** 10.1038/s41419-021-03656-1

**Published:** 2021-04-06

**Authors:** Shoufang Xu, Yilei Ma, Qingchao Tong, Jun Yang, Jia Liu, Yanzhong Wang, Guoli Li, Jin Zeng, Sining Fang, Fengying Li, Xinyou Xie, Jun Zhang

**Affiliations:** 1 Department of Clinical Laboratory, Sir Run Run Shaw Hospital, School of Medicine, Zhejiang University, Hangzhou, Zhejiang, P.R. China; 2 Key Laboratory of Biotherapy of Zhejiang Province, Hangzhou, Zhejiang P.R. China; 3 Department of Cytopathology, Ningbo Diagnostic Pathology Center, Ningbo, Zhejiang P.R. China; 4 Ningbo Medical Center Lihuili Hospital, Ningbo, Zhejiang P. R. China

**Keywords:** Colorectal cancer, Apoptosis, Neddylation, Ubiquitylation

Correction to: *Cell Death and Disease*

10.1038/s41419-021-03557-3 published online 12 March 2021

The original version of this article unfortunately contained a mistake in the legend of fig. 1B. The references in the sentence “The data sets were taken from TCGA^35^ and Hong^36^” were mis-marked and should be corrected to “The data sets were taken from TCGA^25^ and Hong^26^”. The authors apologize for the mistake. The original article has been corrected.

