# Supplementary figures and images for: Cullin-5 neddylation-mediated NOXA degradation is enhanced by PRDX1 oligomers in colorectal cancer
Source: Cell Death Dis. 2021 Mar 12;12(3):265. doi: 10.1038/s41419-021-03557-3 (PMC7954848; doi:10.1038/s41419-021-03557-3)

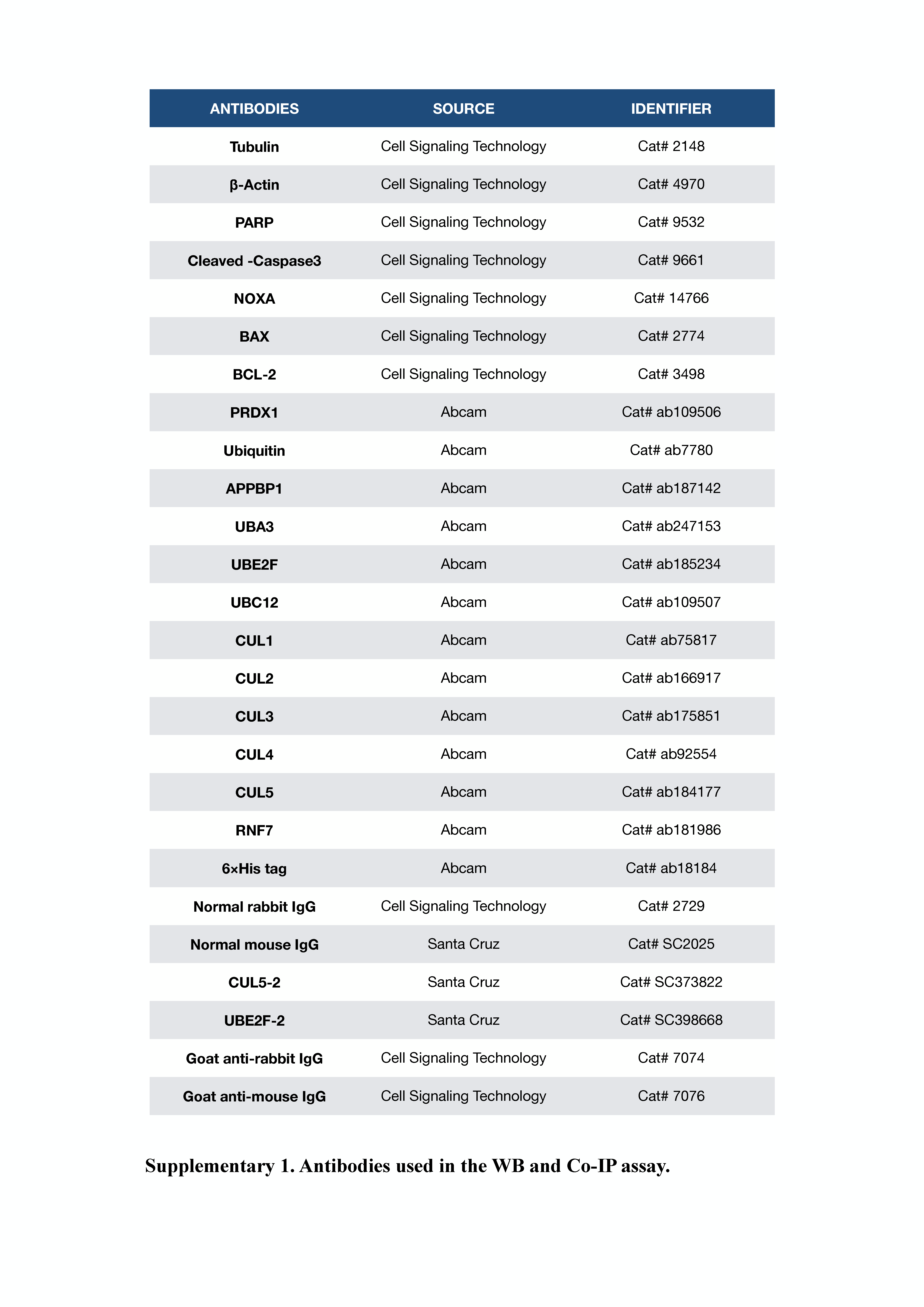

Supplement: Supplementary file 1 — Supplementary 1 [file 41419_2021_3557_MOESM1_ESM.png]

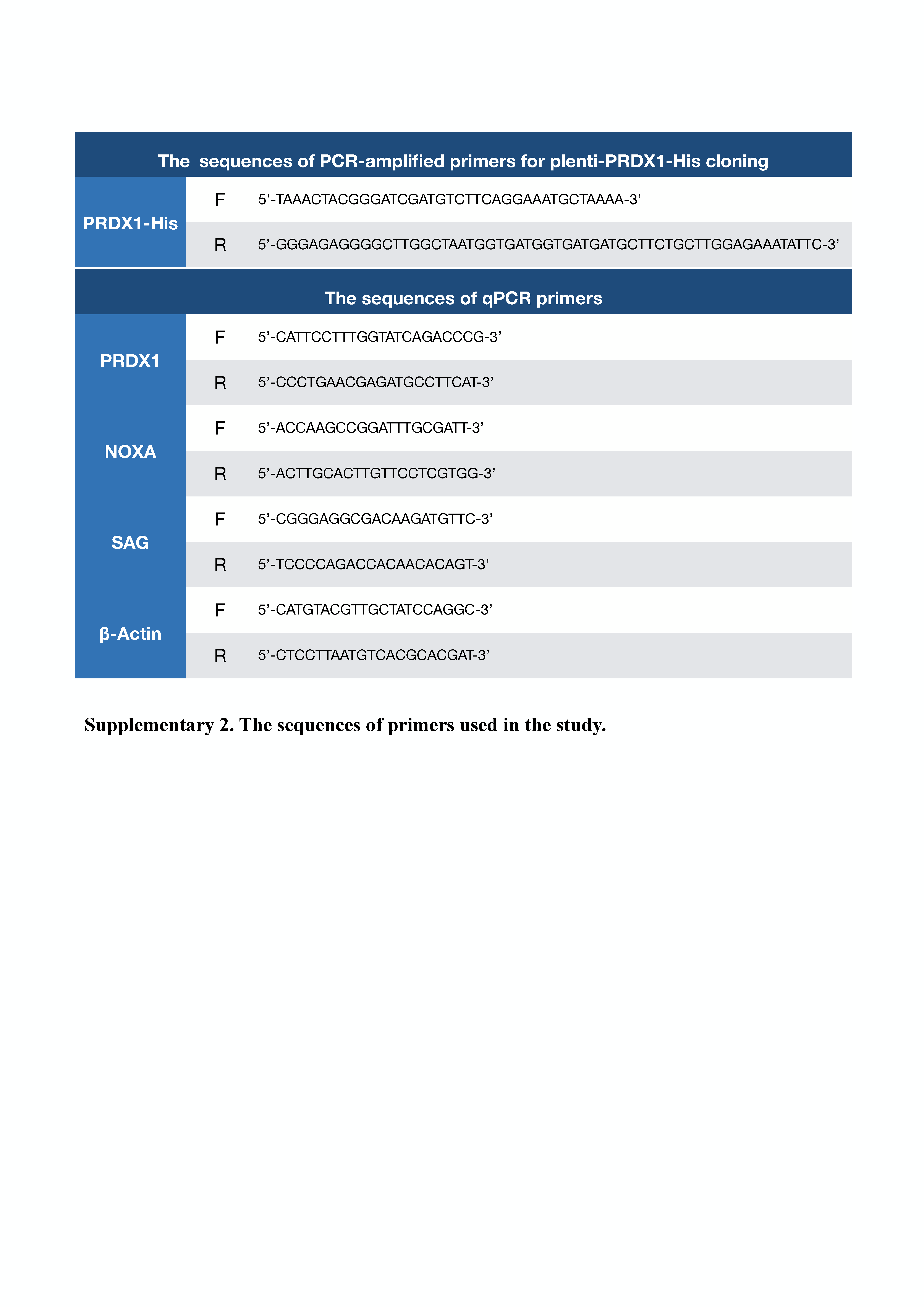

Supplement: Supplementary file 2 — Supplementary 2 [file 41419_2021_3557_MOESM2_ESM.png]

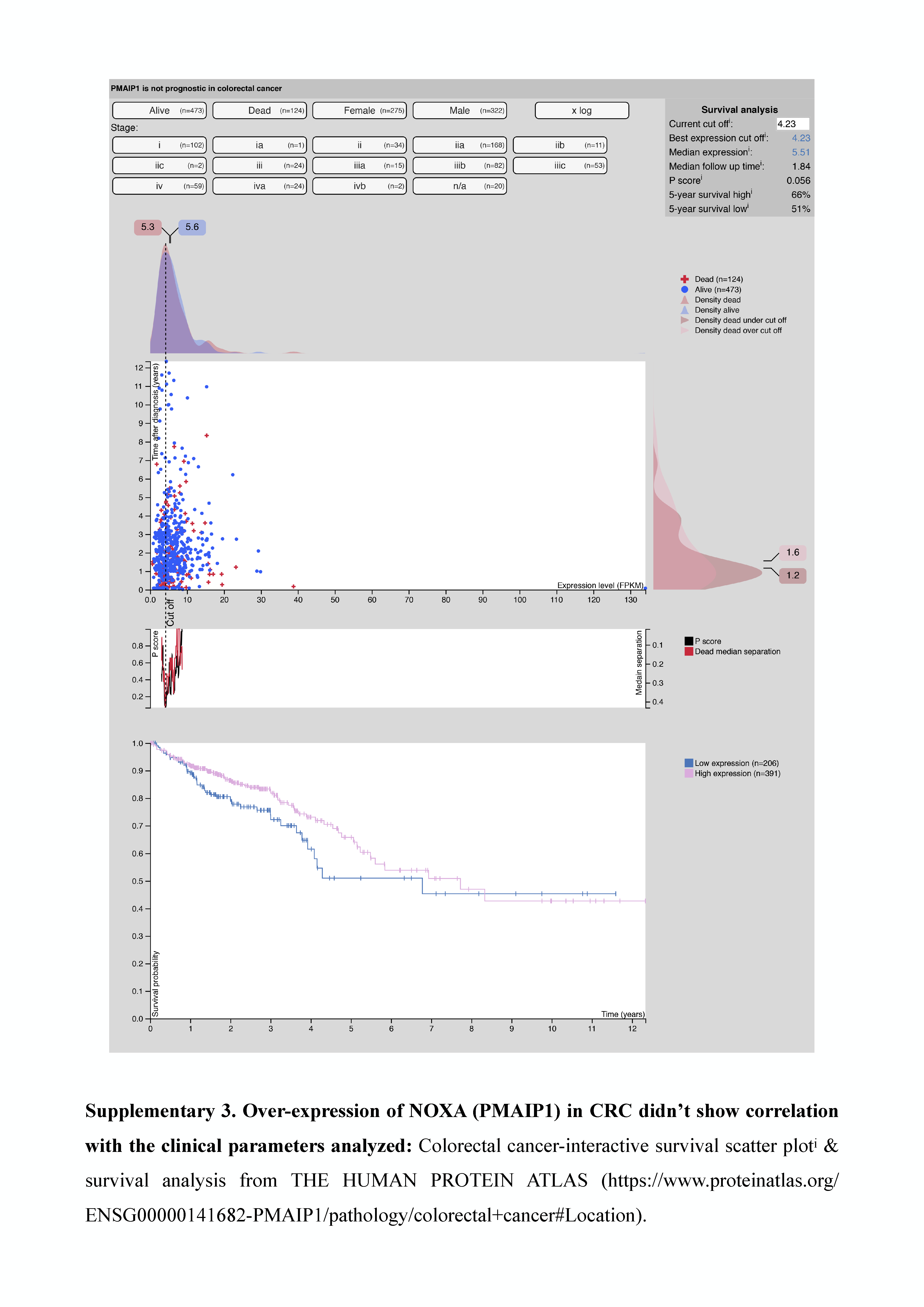

Supplement: Supplementary file 3 — Supplementary 3 [file 41419_2021_3557_MOESM3_ESM.png]

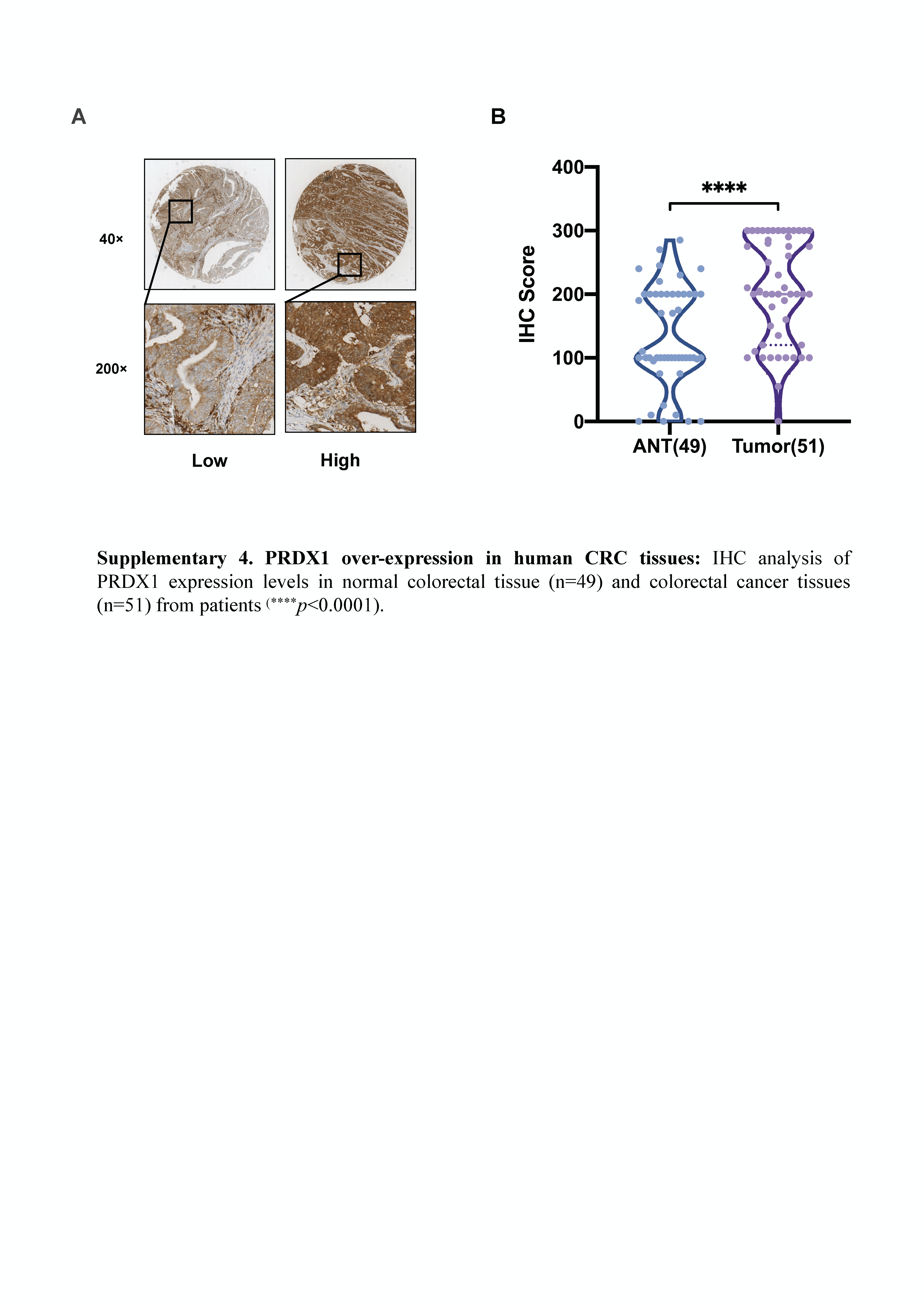

Supplement: Supplementary file 4 — Supplementary 4 [file 41419_2021_3557_MOESM4_ESM.png]

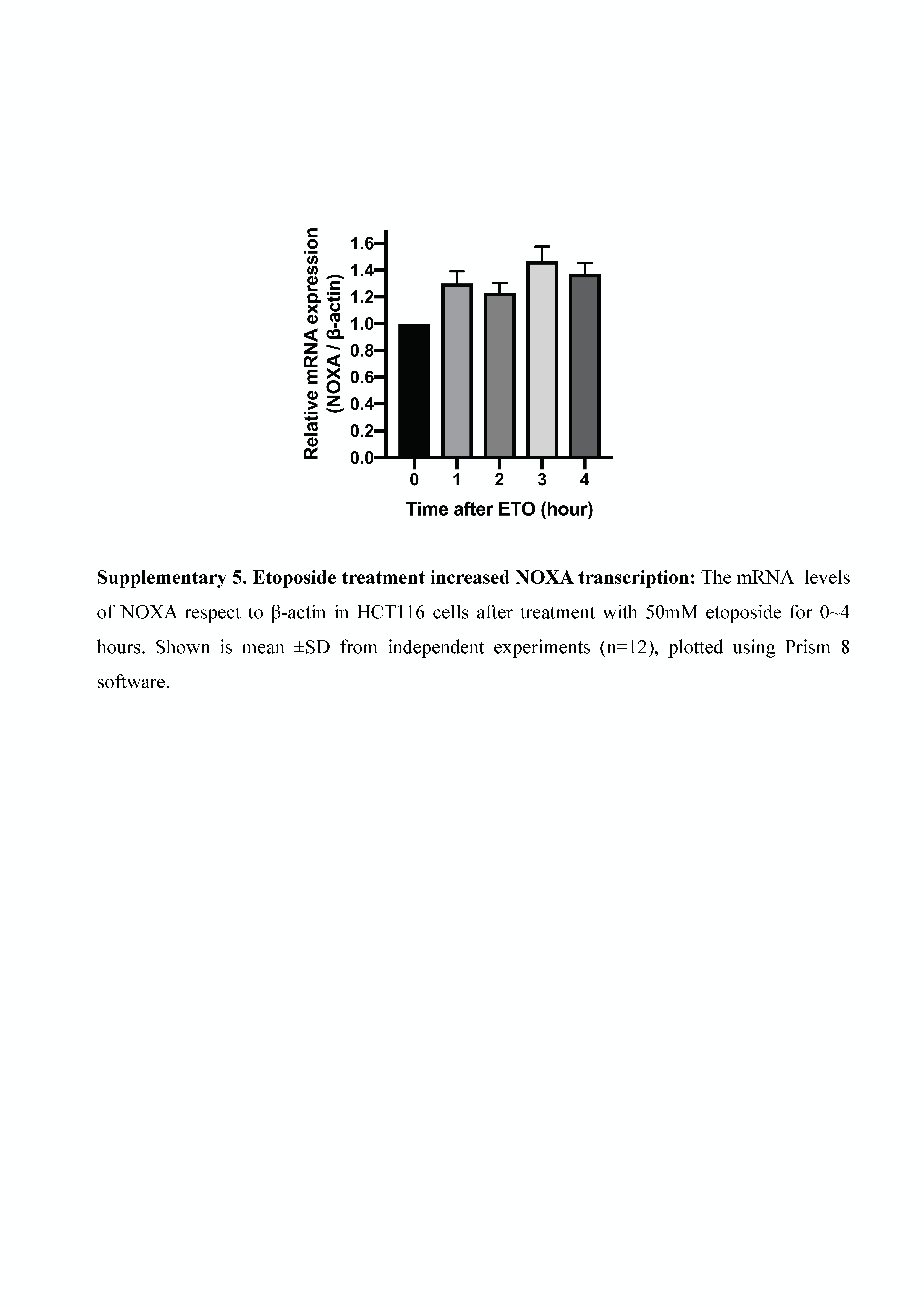

Supplement: Supplementary file 5 — Supplementary 5 [file 41419_2021_3557_MOESM5_ESM.png]

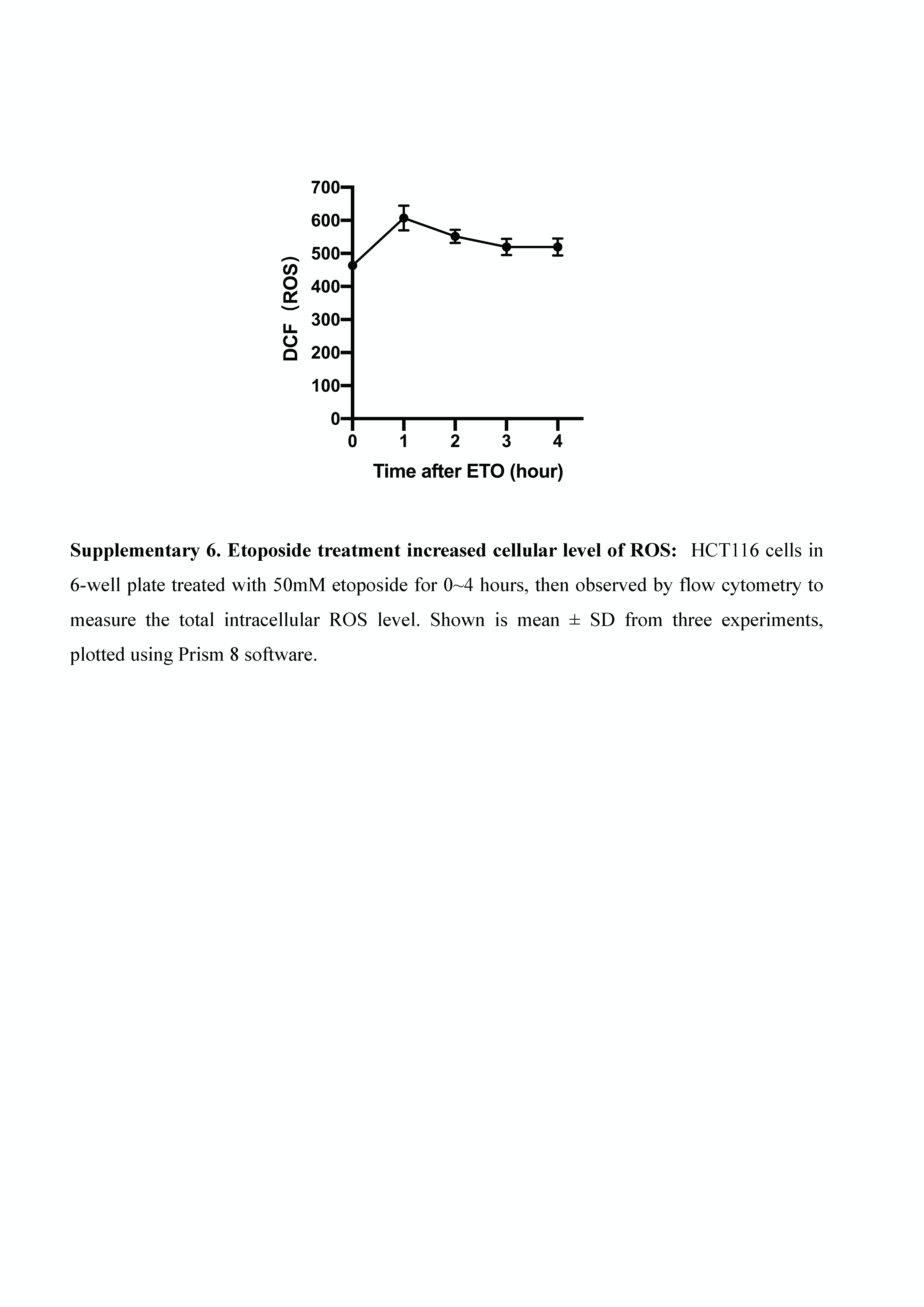

Supplement: Supplementary file 6 — Supplementary 6 [file 41419_2021_3557_MOESM6_ESM.png]

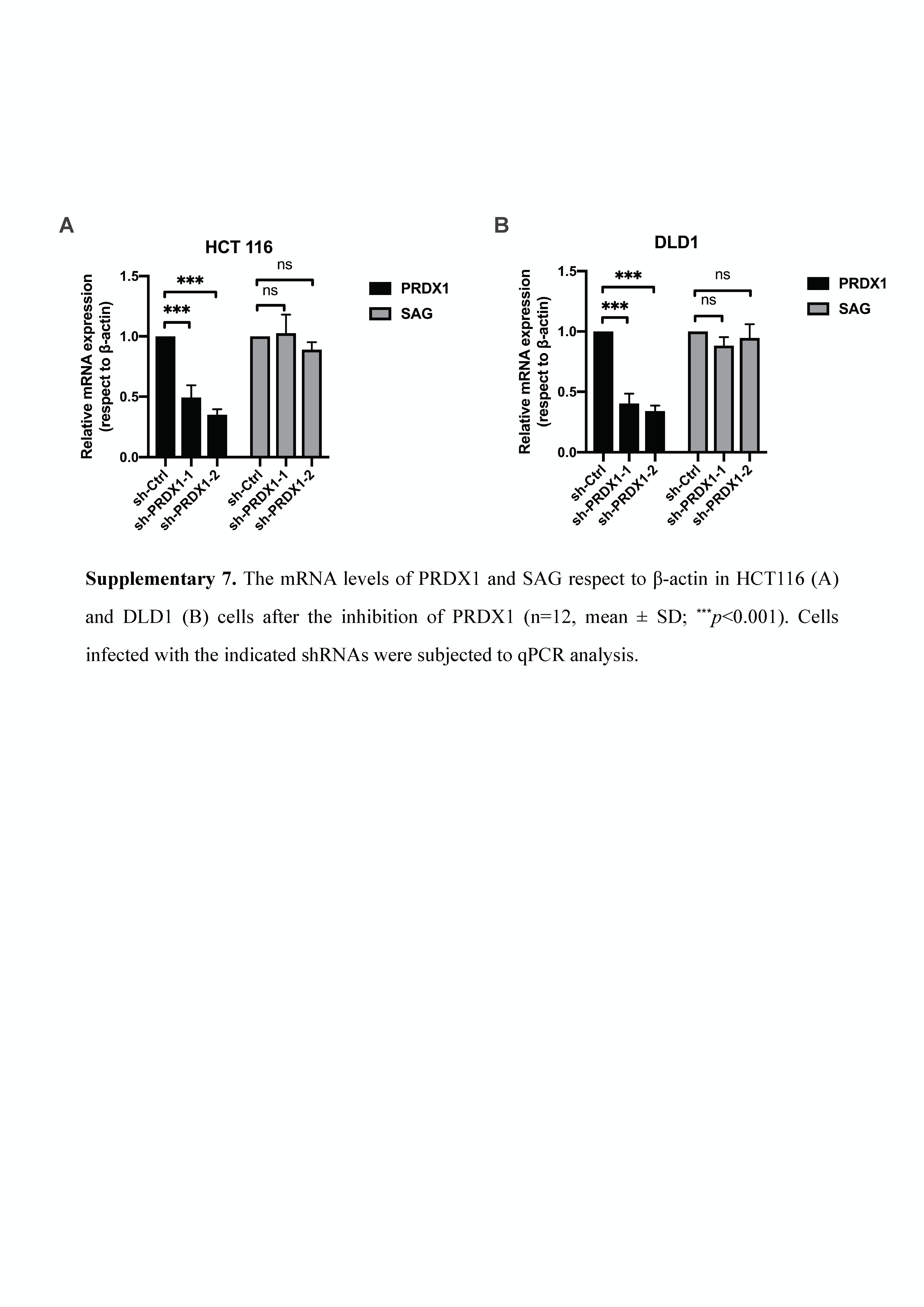

Supplement: Supplementary file 7 — Supplementary 7 [file 41419_2021_3557_MOESM7_ESM.png]

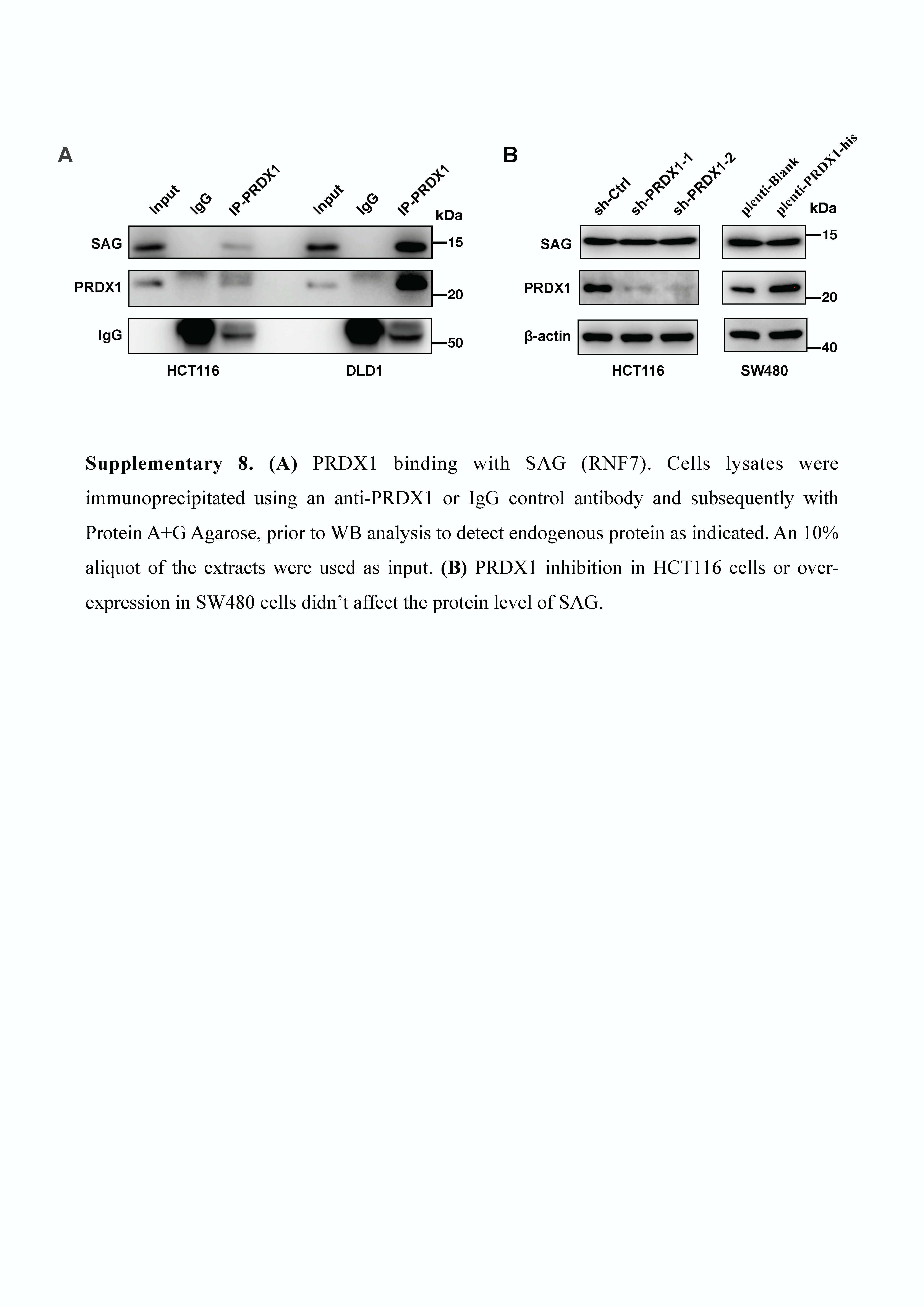

Supplement: Supplementary file 8 — Supplementary 8 [file 41419_2021_3557_MOESM8_ESM.png]

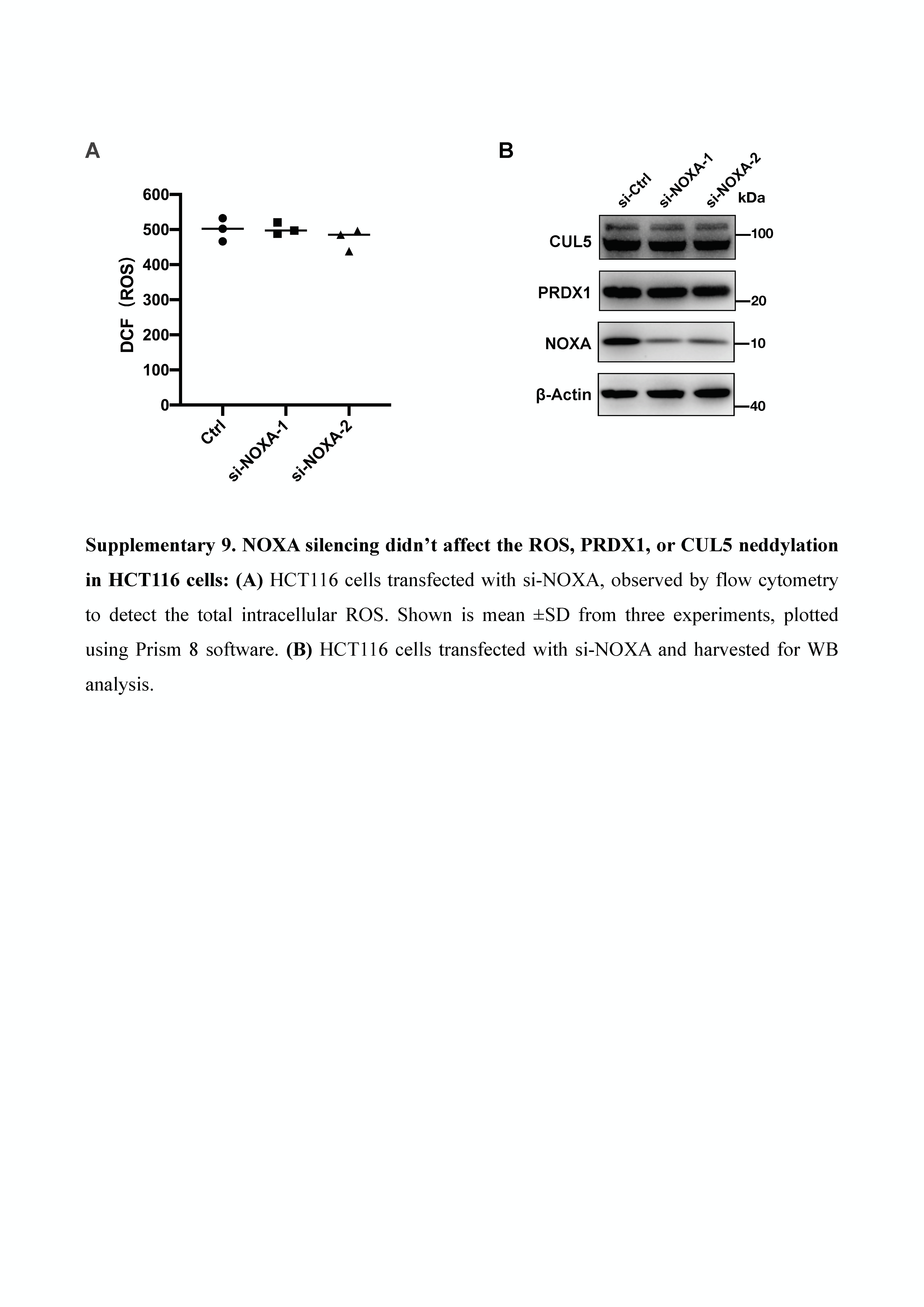

Supplement: Supplementary file 9 — Supplementary 9 [file 41419_2021_3557_MOESM9_ESM.png]
